# A Multi-Scale Entropy Approach to Study Collapse and Anomalous Diffusion in Shared Mobility Systems

**DOI:** 10.3390/e24050606

**Published:** 2022-04-27

**Authors:** Francisco Prieto-Castrillo, Javier Borondo, Rubén Martín García, Rosa M. Benito

**Affiliations:** 1 Facultad de Ciencias, Departamento de Matemáticas, Universidad de Oviedo, Calle Federico García Lorca 18, 33007 Oviedo, Spain; 2 Facultad de Ciencias Económicas y Empresariales (ICADE), Departamento de Gestión Empresarial, Universidad Pontificia Comillas, Calle Alberto Aguilera 23, 28015 Madrid, Spain; jborondo@comillas.edu; 3 Facultad de Informática, Universidad Pontificia de Salamanca, Calle Compañía, 5, 37002 Salamanca, Spain; rmartinga@upsa.es; 4 Grupo de Sistemas Complejos, E.T.S. de Ingeniería Agron., Alim. y de Biosist., Universidad Politécnica de Madrid, Avda. Puerta de Hierro 2-4, 28040 Madrid, Spain; rosamaria.benito@upm.es

**Keywords:** multi-scale entropy, shared mobility systems, super diffusion, death-birth processes, complexity

## Abstract

In this paper, we study the phenomena of collapse and anomalous diffusion in shared mobility systems. In particular, we focus on a fleet of vehicles moving through a stations network and analyse the effect of self-journeys in system stability, using a mathematical simplex under stochastic flows. With a birth-death process approach, we find analytical upper bounds for random walk and we monitor how the system collapses by super diffusing under different randomization conditions. Using the multi-scale entropy metric, we show that real data from a bike-sharing fleet in the city of Salamanca (Spain) present a complex behaviour with more of a 1/f signal than a disorganized system with a white noise signal.

## 1. Introduction

Cities are gigantic systems in which transport networks of matter, individuals and information attempt to self-organise and cooperate to establish certain spatio-temporal patterns so that their inhabitants optimise—at best—their quality of life. In any case, urban environments can be considered machines that adapt their transport networks to maximise global production based on the working patterns of citizens. Under the top–down pressures of both energy efficiency and low greenhouse gas emission policies, transport networks have had to be modified. In particular, the sharing of cars, bicycles or other means of transport in cities, known as shared mobility (SM) systems, is becoming increasingly common. Vehicles are pulled from and returned to a network spatially distributed over the city. Whereas public transport displaces large masses of users at regular timings and fixed locations, SMs operate at a smaller scale allowing higher flexibility. The payback of this elasticity is the injection of different amounts of uncertainty into the system. Therefore, understanding the dynamics of SMs and their impact on mobility patterns at the city scale is a matter of primary importance. This is the main goal of this work.

In the last decades, the problem of mobility has received a lot of attention [1], and universal laws have been proposed: despite diversity in travel history, people follow simple reproducible patterns [2,3]. In particular, bike sharing mobility has been extensively studied since they are sustainable and zero-carbon emission mobility elements [4,5,6,7,8,9,10,11,12,13,14,15].

For example, Borgnat et al. [4] performed a descriptive data analysis of the spatio-temporal patterns of bikesharing in Lyon and developed an aggregate probability prediction by forecasting the number of rentals. They also provided data for the imbalance of the stations network. A trimodal activity of morning, rush hour and evening peaks was found by Austwick et al. [7]. This pattern persists for several cities (London, Boston, Denver, and Washington Minneapolis), which suggests that the trimodal pattern is in fact universal. In their analysis, the authors used the concept of entropy to compare the number of A-B trips with respect to the whole activity—the so-called *trip entropy*—and found that there is not too much variation in the entropy over a day. Interestingly, in some cases (e.g., Minneapolis during weekends), the authors found that self-journeys dominate the whole dynamics. Additionally, the benefits of sharing networks using cab data in NYC were analysed [8]. It was shown that cumulative trip lengths can be reduced at least by 40% when trips are shared. It is worth mentioning that the lack of external action quickly leads to network collapse in terms of depletion/overfull [10]. As the system tends to unbalance, it necessitates the action of an external agent to the system (regulation system) that can operate in times off (static rebalance) or during times on (dynamic rebalance). There is a large amount of related work about how to model and optimize this regulation system [9,10]. Analysing data from 13 stations in London [11], a bimodal pattern for trips that contrasts with the trimodal one reported in [7] was found. This is probably due to the fact that in [11], the authors split the user profiles into registered, unregistered, casual, regular and commuter. We conjecture that the aggregated trip frequencies in [11] would render an additional peak between the two maxima in the bimodal pattern. However, more insight into this topic is left for future work.

Preisler et al. [13] reported the presence of self-organisation patterns when bicycles were forced to wander over the network after encountering full stations. However, the authors did not provide any metrics to quantitatively assess the alleged self-organization. The concept of shareability as the fraction of trips that can be shared was introduced by Tachet et al. [14]. By measuring how shareable an A–B route was in several cities, they found a universal scaling law of shareability. Moreover, the authors provided a simple model to account for such a metric without the need to adjust parameters. Finally, the birth and death model was used to implement dynamic rebalancing for station occupancy in NYC [16].

In a previous work [17], Prieto-Castrillo et al. investigated the conditions for collapse mechanisms of shared mobility systems as perturbed walks in a simplex. In that study, the authors introduced the mathematical apparatus that allowed them to find super-diffusive behaviour in the dynamics of the system. In this new contribution, the authors extended the previous work and analysed the complexity of the system by means of multi-scale entropy and the complexity profile. In addition, this work includes an analysis of the expected collapse times of the system by comparing a mathematical deduction with experimental values. The new results are also validated with empirical data as was done in [17]. In short, this paper introduces novel results by providing analytical limits for system imbalance, comparisons with data and by highlighting the effect of self-journeys (loops) into the dynamics by inspecting the multi-scale entropy and the complexity profile.

The paper is organized as follows: First in Section 2, we formulate the set of all allowable station’s occupancies (state space) in terms of a mathematical simplex. Such a metaphor opens the way to understanding system collapse in terms of imbalance and energy. In Section 3, we use the energy defined before to show how the empirical data follow a typical pattern of super diffusion. We also monitor the effect that loops play on the dynamics of the system. In Section 4, we investigate the complexity of the system under different conditions, using both the multi-scale entropy and the complexity profile metrics. With our model and a stochastic birth–death process, we find in Section 5 the analytical bounds for the collapse of the system. We also check the situation of a real system with respect to the random walk for different levels of randomness. Finally, we conclude the work in Section 6.

## 2. The Whole Picture: Simplex and Microstates

A shared mobility system is efficient, as long as users can access transport units without waiting. To this end, each station must have at least one unit available to start a new journey and an empty place to deposit an incoming vehicle. Our model starts from a fleet of *N* transport units that transit in a network of *D* stations with capacity *C*. The number of vehicles at each station is denoted by the quantities $s_{i}\in N,i\in D$. For example, if we have three stations, the quantities $s_{1}$, $s_{2}$ and $s_{3}$ represent the number of vehicles in each of them, respectively, and the state of the system will be $s=(s_{1},s_{2},s_{3})$. In this first approach, we consider the size of the fleet fixed. This way, we define the state space Ω as the simplex:

(1) $$\Omega =\{s\in N^{D}\;|\;\sum_{i=1}^{D}s_{i}=N,0\leq s_{i}\leq C\}$$

with s being the D-dimensional occupancy (state) vectors of the system. This simplex is a hyperplane of dimension D−1. Given *D* stations with capacity *C*, the number of possible arrangements for *N* units—see Supplementary Material—is given by Equation (2):

(2) |Ω|=∑j=0D(−1)jDjD+N−1−j(1+C)N−j(1+C).

**Figure 1:**
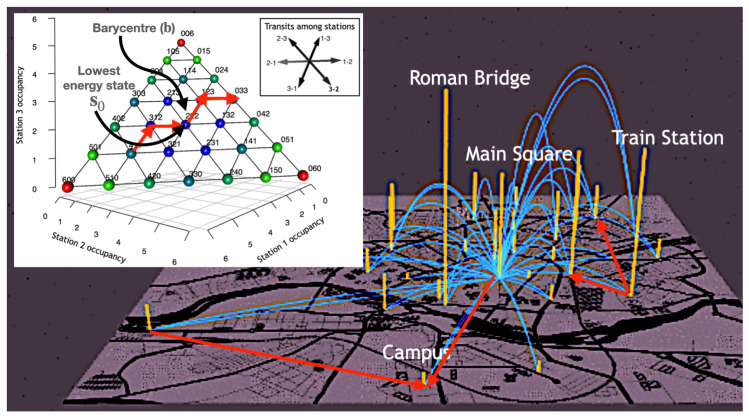
Schematic of the system dynamics. Vehicle flows between stations (blue arcs) can be mapped to a walk in the simplex (inset). As these flows are not symmetrical, the system is destabilising. This figure also shows the loops (orange bars) that produce no energy change in the simplex since adding and subtracting one unit to the same station produces no net change in the system state s. INSET: Walks in the simplex. A toy state space with only D=3 stations, N=6 units with capacity C=6. Each vertex represents a possible state of the network and labels show the occupancies of each station. Colours are proportional to the distance from each point to the barycentre of the simplex located at (3,3,3). The lowest energy state $s_{0}=\left(2,2,2\right)$ is also indicated. The possible directions in which states can transit are shown by the black arrows in the inset. For example, 2−3 means moving a unit from station 2 to station 3. This way, starting at state s=(1,3,2), the corresponding flow would take us to state $s^{\prime }=\left(1,2,3\right)$. A path of three possible transitions is represented in the inset as red arrows.

For example, for the toy model consisting of 6 units and 3 stations with capacity C=6, there are 28 possible arrangements (Figure 1 inset). Since this number grows exponentially with *D* and *N*, the state space must be explored by heuristic methods.

Trips originated at station *i* with occupancy $s_{i}$ to station *j* with occupancy $s_{j}$ are then modelled as directed links (i,j)∈D×D in the simplex (Figure 1 inset). This way, a trip increases the number of units at destination *j* by one unit while it decreases the available vehicles at the origin *i* by one unit. The corresponding change of system state in the simplex can be described as

(3) $$s^{\prime }=s+\Phi \left(i,j\right)$$

where $\phi_{k}\left(i,j\right)\equiv \left(1-\delta_{ij}\right)\left(\delta_{jk}-\delta_{ik}\right)$ are the components of the D-dimensional flow vector Φ(i,j). The transition is defined as valid (possible) when the two following conditions hold:

1. $s_{i}>0$ (there is at least one unit at the origin);
2. $s_{j}\leq C$ (the destination is not overfull).

Note that a valid transition corresponds to a mapping between one state s and another state $s^{\prime }$ in its neighbourhood in the simplex $U_{s}$. This neighbourhood is defined as the points that are at a distance less or equal to $\sqrt{2}$:

(4) $$U_{s}=\{s^{\prime }\in \Omega \;|\;||s-s^{\prime }\left|\right|\leq \sqrt{2}\}.$$

For example, starting from the state s=(0,6,0), there are two valid transitions. One is (i,j)=(2,1) (going from station 2 to station 1), which corresponds to $s=\left(0,6,0\right)\to s^{\prime }=\left(1,5,0\right)$, and the other one is (i,j)=(2,3) (going from station 2 to station 3), which corresponds to $s=\left(0,6,0\right)\to s^{\prime }=\left(0,5,1\right)$. Our model also captures non-possible trips since a transition like (i,j)=(1,2) would pretend to bring a unit from station 1 (that is empty) to station 2 (which is also full). This non-possible transition would lead to a state outside the simplex as can be seen in the Figure 1 inset. Therefore, the simplex has the property of being a set of valid states in which the motions are continuous. It should be remarked that only a certain subset of (i,j) transitions are allowed due to the boundary limit states.

Notice also that states located far from the simplex centre will tend to fall into imbalance more easily. This is because the vehicle system is balanced when station occupancy is close to 50%. This maximizes the likelihood that units will be available and that there will be space to leave a unit. This model allows us to quantify the imbalance of the state s as its distance to the barycentre of the simplex. We can further exploit the analogy with a physical system by defining the energy of a state as $H\left(s\right)=||s-{b||}^{2}$, where b=(C/2,C/2,…,C/2) is the simplex barycentre. Note that b need not be a state of the simplex (see Figure 1 inset). The idea is that points near the barycentre are more balanced and therefore have lower energy. Note that for a valid transition (i,j) we have an energy change:

(5) $$\Delta H=H\left(s^{\prime }\right)-H\left(s\right)=2\left(1-\delta_{ij}\right)\left(1+s_{j}-s_{i}\right).$$

In this setting, the equi-energetic surfaces correspond to states lying at equal distance from the simplex barycentre. This way, self-journeys or loops i=j have zero energy change.

Moreover, through the equivalence relation $s∼s^{\prime }\Leftrightarrow H\left(s\right)=H\left(s^{\prime }\right)$, it is possible to partition the simplex into macro-states. These macrostates are thus the equal-balanced surfaces in the quotient set Ω/∼ as defined in [18]. A schematic of the system dynamics is shown in Figure 1: flows among stations (arcs) are mapped to a walk in the simplex (inset). Since these flows are not symmetrical, the system tends to destabilize. This figure also shows the loops that have no energy change in the simplex depicted as vertical bars.

The lowest energy state $s_{0}$ is the one closest to the simplex barycentre: $s_{0}=\arg\min H\left(s\right)$. Hence the lowest energy of the system is

(6) $$H^{min}=H\left(s_{0}\right)=D{\left(\left\lfloor C/2\right\rfloor -C/2\right)}^{2}.$$

Since $s_{0}$ is the closest state to b, this configuration corresponds to balancing the load of all stations in the network with a number of units close to C/2.

In the opposite case, the imbalance occurs when stations are filled to their limit and others remain empty. By performing the integer division N=QC+r for Q=0,…,D and r=0,…,C−1, a typical unbalanced state will be of the form given in Equation (7).

(7) $$s=(\underset{Q\;times}{\underbrace{C,C,\ldots ,C}},r,\underset{D-Q-1\;times}{\underbrace{0,0,0,\ldots }})$$

This corresponds to *Q* stations with their maximum occupancy, *C*, (D−Q−1) stations being completely empty and one station with occupancy *r*, being the energy of this state:

(8) $$H\left(s\right)=\frac{DC^{2}}{4}-r\left(C-r\right).$$

Note that permutations among the *s* components produce the same energy. The maximum is obtained for states in which r=0: $H^{max}=DC^{2}/4$. Note that by construction, the states closest to collapse are on the simplex boundary. These states are only one transition away from producing the collapse of the system.

Notice also that the simplex model only gives sufficient conditions for the system to “collapse”, where collapse is understood to be a lack of service due to a station being either empty when one intends to purchase a vehicle or full when one intends to deposit their vehicle. It is clear that the system can collapse for many other reasons not considered in our model (e.g., closing of stations or other enforced external actions).

## 3. Asymmetry and Super Diffusion: The Effect of Self-Journeys (Loops)

**Figure 2:**
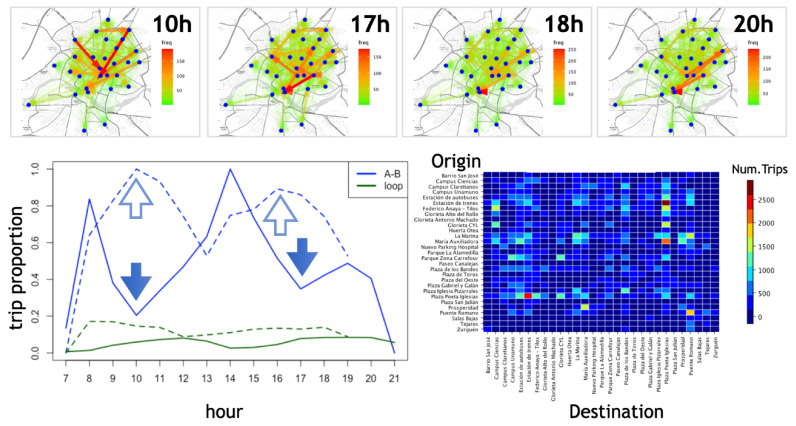
Mobility patterns for a fleet of 29 bike sharing stations in the city of Salamanca. **Top**: on-map representation of aggregated trips at different hours. **Bottom left**: evolution of the number of trips (blue) and loops (green) observed during workdays (full line and filled arrows) and weekends (dashed line and hollow arrows). Notice the trimodal behaviour of the A–B trips during workdays, compared to the bimodal pattern during the weekend. **Bottom right**: matrix-like representation of the frequency of trips highlighting a large asymmetry present in the empirical trip probabilities $p_{ij}$.

Based on the model defined above, we inquire how spatio-temporal asymmetries in the distribution of stations and users’ schedules create different mobility patterns and how these latter contribute to the overall system collapse. To do so, we start from a set of 17,000 trips stored as time-stamped origin–destination records. The data were collected in the period 2014–2017 in the city of Salamanca (Spain). There are 29 stations and as found in [7], we observe in Figure 2 a trimodal pattern of mobility correlated to the daily working activity.

Notice however that the trimodal pattern of A–B trips during workdays shifts to a a bimodal pattern on the weekend. Additionally, self-journeys of the form A–A (in this paper referred to simply as 1-trip loops or loops) have a bimodal pattern for both workdays and weekends. The reason is that loops have a different dynamic than A–B workday trips but are not so different than A–B journeys during weekends. This is likely caused by different user profiles who have different priorities (e.g., on weekends, people do not need to commute at lunchtime and so the central peak disappears).

A global portrait of the movements at different times can be obtained by monitoring the flows at different timestamps (Figure 2 Top). To do this, at a given time, we extract the trips and sort them by frequency. Then, we generate a uniform distribution between 1 and the maximum number of trips for each hour. Then, we choose the paths with a frequency greater than 50% of the paths in that hour (in Figure 2, we only present the hours with the highest incidence as the others have less striking patterns). Loops represent only 6.47% of the trips, but as we will see below, they play an important role. Notice the organized activity at 20 h compared to the more chaotic-like pattern at 17 h.

**Figure 3:**
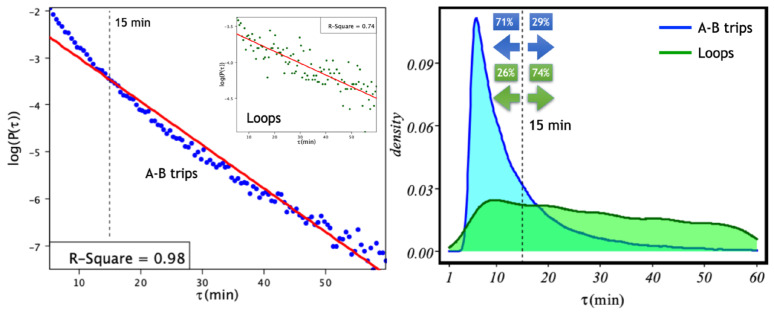
Inter-arrival times (IAT) for A–B trips and loops. Left: Log–log plot of the distribution and the corresponding fit (red) to a Poisson distribution for both A–B trips and loops (inset). The fitted μ values are μ=0.0925 min ≈ 1 event per 10 min (A–B trips) and μ=0.016 min ≈ 1 events per hour (loops). Right: IATs probability density for all trips (blue) and loops (green). The dotted line shows the separation of the two time scales in the case of A–B trips: one for trips lasting <15 min and one for longer trips. This effect is not observed for loops. Finally, in the distributions of IATs, we show the percentages of short (<15 min) and longer (>=15 min) trips.

In Figure 3, we show the inter-arrival time (IAT) distribution for both A–B trips and loops for the fleet of 29 bike-sharing stations in the city of Salamanca. Firstly, we notice that for the case of A–B trips (Figure 3 left), a fit to a Poisson distribution P(μ)=exp(−μτ) seems to reveal the existence of two time scales: one corresponding to trips shorter than 15 min and another scale corresponding to trips of larger times. This effect is not observed for self-journeys (Figure 3 left inset), although in this case, the fitting is weaker ($R^{2}=0.74$). To further clarify whether loops are responsible for the two-scales effect not captured by the Poisson model in Figure 3 right, we analyse the distributions of both types of trips. There, we notice that loops have a much longer tail than A–B trips and that most self-journeys (74%) correspond to longer times. This rules out the possibility that loops are responsible for the two observed time scales. Although it is very interesting to delve into the reasons for this division of scales, this would take us too far in this first work, and we leave its analysis for future work. The fitted rates for A–B trips and loops are μ=0.0925 min ≈ 1 event per 10 min (A-B trips) and μ=0.016 min ≈ 1 events per hour (loops).

On the other hand, loops produce no net change in the balance of units in the stations. Thus, loops must play an important role in the dynamics of the system. Clearly, the complexity of the dynamics arises from the spatial distribution of the stations, the temporal patterns of the users and the correlation of these two factors with the number of loops. In this respect, we ask three questions: (1) how to compare the movements in the simplex with respect to random walks, (2) how to characterise the complexity of the system, and (3) how to quantify the expected collapse time. Let us first look at what a diffusion process in the simplex looks like.

The m−length walk in the simplex is constructed by iterating Equation (3) for *n* steps:

(9) $$s_{n}=s_{0}+\sum_{m=1}^{n}\Phi_{m}$$

with $\Phi_{m}$ being simple random variables taking values Φ(i,j). By assuming $s_{0}=b$, energies can be calculated as follows (If the first state is not exactly the barycentre, there is only one correction as an additive constant):

(10) $$H_{n}=\sum_{m=1}^{n}{\left|\Phi_{m}\right|}^{2}+2\sum_{a>b}^{n}\Phi_{a}\cdot \Phi_{b}.$$

However, noticing that $|\Phi_{m}{|}^{2}=2,\forall m$—this holds from the definition of Φ(i,j)—the mean value of the energy is as follows:

(11) $$<H_{n}>=2\left(n+\sum_{a>b}^{n}<\Phi_{a}\cdot \Phi_{b}>\right).$$

**Figure 4:**
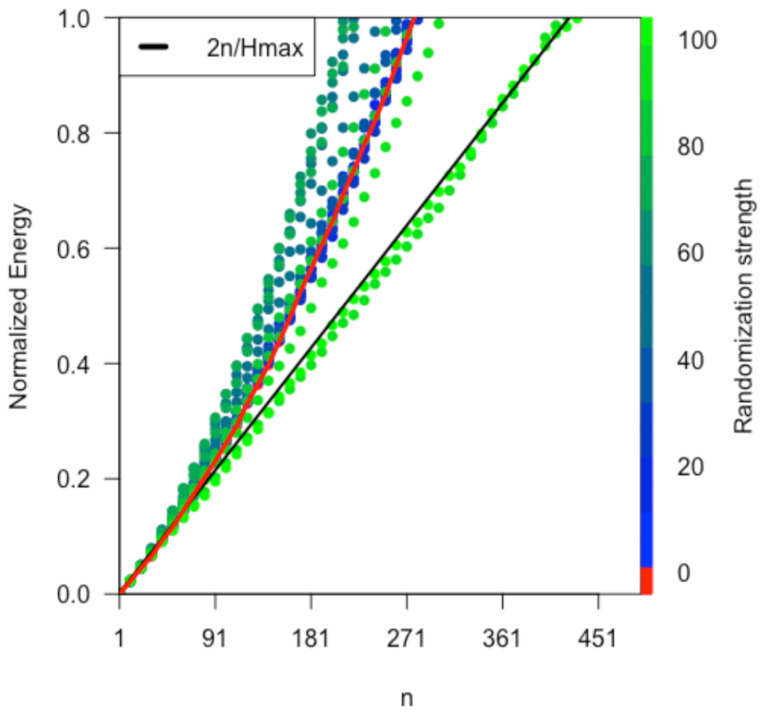
Super diffusive behaviour. Normalized energy—Equation (11) (re-scaled between 0 and 1)—as a function of the number of steps *n* for different randomisation strengths. The maximum randomisation limit is also shown in the black line.

The super-diffusive term will be 0 for purely random motions. In this case, we obtain $<H_{n}>=2n$. However, the presence of non-symmetric flows renders higher diffusive behaviour. To check this, we compare the diffusion in the observed trips with random walks by simulating walks starting from a state near b and driven by the empirical probabilities $p_{ij}$ corresponding to a i→j trip. Furthermore, we increasingly randomize the $p_{ij}$ weights and monitor the effect. We then plot the normalized energy in the simplex for each iteration as shown in Figure 4. We describe this process in detail in the Supplementary Material.

**Figure 5:**
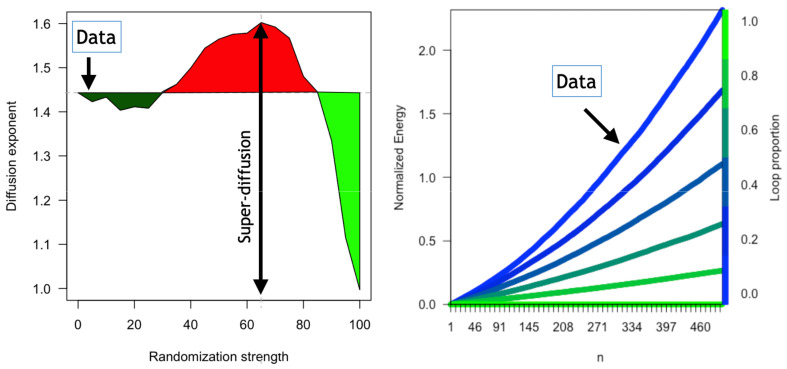
Super diffusion and loops. **Left**: diffusion exponent in Equation (12) as a function of the randomization strength. **Right**: Effect of loop trips in system diffusion: as the number of loops increases, the system becomes less and less super-diffusive. Each curve represents the normalized energy—Equation (11) (re-scaled between 0 (blue) and 1 (green)) as a function of the number of steps *n* obtained by Monte-Carlo walks in the simplex.

By fitting the curves for each power law of the form,

(12) $$<H_{n}>∼n^{\gamma }$$

where *n* is the number of steps, we have also calculated the diffusion exponents (Figure 5 left) for each randomization level. Note that, as we increase the percentage of random trips, three phases appear:

1. <30% random trips: diffusion is less rapid than that found in the data (γ=1.44).
2. 30–90% random trips: the super-diffusive regime spreads faster than that observed in the data and peaks at 65% for γ=1.60.
3. >90% random trips (near total random): the super-diffusion drops abruptly, and the system starts to diffuse as a random walk in which the normalized energy (unbalance) grows linearly with *n* and γ=1.

On the other hand, in Figure 5 right, we monitor the normalized energy as the proportion of loops in the data grows. As noticed, loops slow down the diffusion and when all trips become loops, there is no diffusion at all. Figure 5 also shows how the system naturally overdiffuses for real data and that in general external mechanisms are necessary to rebalance the system, as it is common in shared mobility.

The asymmetric drift present in the empirical $p_{ij}$ (Figure 2 bottom right) causes the observed super-diffusion, and we conjecture that the macroscopic effect of this mechanism must also have an origin in the long correlations caused by interactions within the system.

## 4. Measuring System’s Complexity: Multi-Scale Entropy and Complexity Profile

To gain more insight into the cause of the observed anomalous diffusion, we quantitatively characterise the complexity of the system. The long-range correlations create a persistence of structures on a temporal scale. Firstly, Refs. [19,20] propose the approximate entropy (*ApEn*) as a measure of changes in the complexity of a system. This metric offers greater robustness than others previously used, and requires a much smaller number of observations. *ApEn* measures the (logarithmic) likelihood that runs of patterns that are close remain close upon next incremental comparisons. For ordered series, a value of *ApEn* close to 0 means that patterns persist on an immediately larger scale, which is typical of a deterministic system.

Later on, [21] extended the *ApEn* metric to sample entropy (*SampEn*) from shortcomings observed in heart rate dynamics. This new measure avoids self-matching in the patterns, resulting in a more reliable estimation, especially when the number of samples is small.

Following the notation of [19], let us consider *N* temporally ordered observations with points u(1),u(2),…,u(N). The idea now is to form vectors with *m* consecutive points, starting from a position *i* in the series: x(i)=[u(i+k):0≤k≤m−1], with 1≤i≤N−m+1. Then, for each *i*, given a threshold *r*, the following quantities are computed:

- $B_{i}$: number of vectors x(j) such that the distance d(x(i),x(j)) lies below threshold *r*.
- $A_{i}$: same as $B_{i}$ but using m+1-sized vectors instead of vectors of length *m*.

Then, according to [21], we define the sample entropy, sampEn, as

(13) $$sampEn\left(m,r\right)=-\log\left(\sum_{i=1}^{N-m}A_{i}/\sum_{i=1}^{N-m}B_{i}\right).$$

Notice that *sampEn* is just the negative logarithm of an estimate of the conditional probability of a match between m+1 vectors given a match of the *m*-sized vectors. Thus, the *sampEn* searches for the probability of a larger pattern, given a pattern of order *m*. Usually, the values m=2 and r=0.2 are used.

It is worth mentioning that the concept of sampEn has been extended to other fields, such as, for example, to the study of physiological time series [22,23] (a systematic review of the multiscale entropy algorithm and its variants is given in [24]). The authors identified the need to introduce a complexity metric more appropriate to biological systems in which a loss of complexity may be a generic feature of pathologic dynamics. This new measure would take into account the fact that complex systems might process information differently depending on the scale. The idea is to quantify the information conveyed over multiple scales. To this end, multi-scale entropy (MSE) [22] can be defined as the computation of *SampEn* for different coarse-grained versions of the series. That is, if we have scales $\tau =1,2,\ldots \tau_{max}$, we create the vectors

(14) $$y_{j}^{\left(\tau \right)}=\frac{1}{\tau }\sum_{i=(j-1)\tau +1}^{j\tau }u\left(i\right),\;j=1\ldots \left\lfloor \left(N/\tau \right)\right\rfloor .$$

As it is well known, chaotic signals [25,26] usually have a memoryless white noise pattern. However, complex systems often present series with 1/f-type noise that manifests correlations between spatio-temporal scales. The authors of [22] use the MSE and find that the multi-scale entropy of arterial fibrillated patients (as in the case of white noise signals) drops rapidly with increasing scale, but the entropy of healthy individuals (such as 1/f noise) remains almost constant across different scales. This behaviour makes the MSE a more reliable metric for identifying complexity than the simple computation of *SampEn* at one particular scale.

In our case, instead of a numerical time series, we have a chain of source–destination pairs of the form $\left\{u\left(n\right)=\left(A_{n},B_{n}\right):n=1,2,\ldots \right\}$. Hence, we must modify the sums of Equation (14). A convenient way to perform this aggregation is to choose the most frequent (i.e., the mode) trip A→B in the corresponding portion of the coarse-grained time series. Thus, we implement MSE as follows:

(15) $$y_{j}^{\left(\tau \right)}=mode\left[u\left(i\right):i=\left(j-1\right)\tau +1\ldots j\tau \right],\;j=1\ldots \left\lfloor \left(N/\tau \right)\right\rfloor .$$

In addition, to calculate the inter-path distances required to compute the *SampEn*, we use the Jaccard distance given by

(16) $$d\left(u\left(i\right),u\left(j\right)\right)=1-\frac{|\tilde{u}\left(i\right)\cap \tilde{u}\left(j\right)|}{|\tilde{u}\left(i\right)\cup \tilde{u}\left(j\right)|}$$

where $u\left(i\right)=\left(A_{i},B_{i}\right)$ and ${\tilde{u}}_{i}=\left\{A_{i},B_{i}\right\}$. Notice that with this definition, trips (i.e., loops) of the form A→B→A have distance 0, while the distance between trips (A,B) and $\left(A^{\prime },B^{\prime }\right)$ is 1. In the Supplementary Material, we show the pseudocode for the explicit construction of the *SampEn* and MSE metrics, respectively.

**Figure 6:**
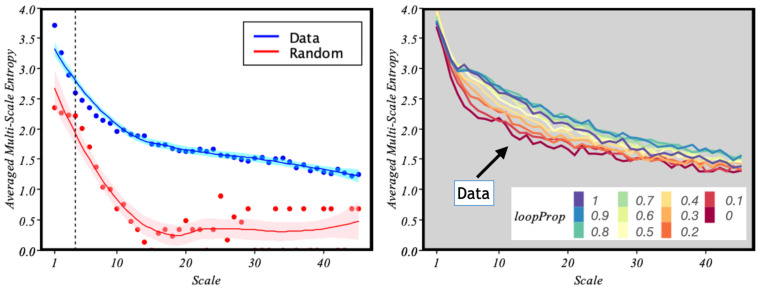
Multi-scale entropy. **Left**: comparison between the MSE of the real data and the simulated random walk. The vertical black dotted line represents the closest point between the real data and the random version. Each data point represents the averaged MSE of 1000 samples. We also show the fitted lines obtained with local polynomial regression (shaded regions representing 95% confidence level intervals). **Right**: MSE obtained under different data-rewiring conditions—with probability loopProp randomly selected A→B trips in the data are transformed into loops A→A. The scale axis refers to the time coarse-grain level used in the computation of the MSE.

In Figure 6, we present the MSE of the data against a randomized situation in which A→B trips are chosen randomly for parameters m=2 and r=0.5. Note that the MSE soon drops rapidly for the random case, but it decreases smoothly in the case of the empirical data series. This effect was observed by Costa et al. [22] when they compared the MSE for white noise and 1/f noise.

In particular, notice also (Figure 6 left) how for a scale around 4, which, according to the event rate μ≈ one event every 10 min (found in Section 3), the MSE of the data is close to that of the random case. Such a scale roughly corresponds to 40 min. From that threshold, a random system would quickly lose information between time scales. However, in the case of urban mobility, the information transmitted between scales is maintained, which is a proxy for self-organisation and complexity.

Furthermore, in Figure 5 (right), we saw that the loops have a stabilising effect by decreasing the energy of the system. Based on Figure 6 right, we see that this result is consistent. Indeed, if we gradually introduce artificial loops into the data, we see that in general, increasing the number of loops makes the behaviour of the system more correlated. Interestingly, the maximum number of loops— loopProp=1—does not make the system more organised, as there seems to be a threshold at loopProb=0.9.

**Figure 7:**
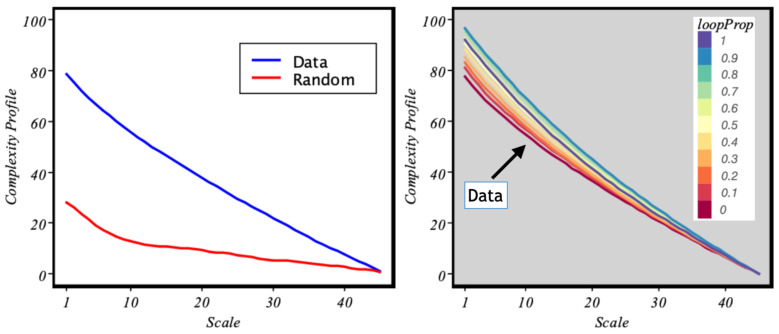
Complexity profile. **Left**: comparison between the real data and the simulated random walk. **Right**: profile obtained under different data-rewiring conditions, with probability loopProp randomly selected A→B trips in the data are transformed into loops A→A. The scale axis refers to the time coarse-grain level used in the computation of the MSE.

Finally, we will use a concept that has proven to be useful for characterising complex systems: the complexity profile [27]. Given a metric of complexity—in our case, we postulate the MSE as a good candidate—the complexity profile simply plots the value of the metric as a function of scale. A complex system is expected to maintain high values of the metric for large scales. Here, we define our complexity profile as the area under the curve (AUC) of the MSE. Consequently, the complexity profile of the data drops much more smoothly than the randomized samples as shown in Figure 7.

In Figure 7, we present the complexity profile. First of all, we can see how this metric is able to separate more clearly the complex behaviour from that of an equivalent random system, as it is more robust under noise Figure 7 left. On the other hand, the complexity profile is also consistent with the role that loops play in the system (right). Self-journeys contribute to increasing the long-range correlations. However, they do so in a way that decreases diffusion and therefore contributes to stabilising the system.

## 5. System’s Collapse and Mean Absorption Times

### 5.1. System’s Collapse

**Figure 8:**
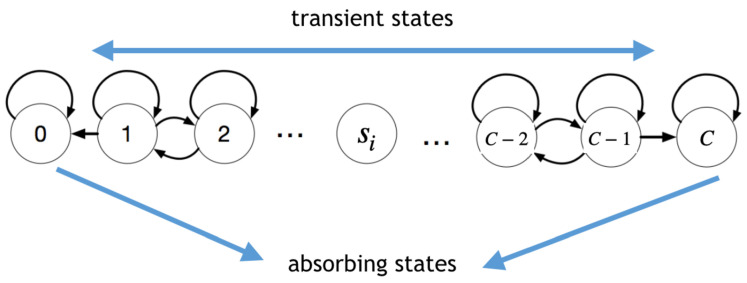
Birth and death model for imbalance. Consider a generic station *i* with occupancy $0\leq s_{i}\leq C$. When some unit travels from station *i* to station *j*, the value of $s_{i}$ decreases by one unit with probability $p_{ij}$. Conversely, $s_{i}$ grows by one unit with probability $p_{ji}$ when a transition (j,i) has occurred. Loops make $s_{i}$ remain constant with probability $l_{i}$. In this chain, the states 0 and *C* are absorbing states because once one of these states is reached, the system collapses.

To answer the last question posed above, let us now analyse in detail the conditions of collapse. In particular, how likely collapse is as the system evolves from an equilibrium situation $s=s_{0}$. The system fails when at least one station is full (no space to leave a vehicle) or empty (a user will have to wait). This process can be understood as a birth–death chain with two reflecting boundaries as shown in Figure 8, where the chain goes from 0 to *C*, and its transitions are *p* (birth), *q* (death) and *l* (loop). In this model, a generic station *i* with occupancy $0\leq s_{i}\leq C$ decreases by one unit with probability $p_{ij}$ when there is a transition (i,j) with j≠i. In this setting, $s_{i}$ grows by one unit with probability $p_{ji}$ when a transition (j,i) has occurred. This way, for each dimension (station) *i*, the evolution of its occupancy $s_{i}$ is driven by a stochastic process determined by the transition probabilities: (1) birth: $p_{i}=\sum_{j\neq i}p_{ji}$, (2) death: $q_{i}=\sum_{i\neq j}p_{ij}$ and (3) loop: $l_{i}=1-p_{i}-q_{i}$. The tridiagonal transition matrix of dimension (C+1)×(C+1) in this birth–death process for $s_{i}$ is

(17) $$M=\left[\begin{array}{ccccc} l_{i} & p_{i} & 0 & ... & 0\\ q_{i} & l_{i} & p_{i} & ... & 0\\ ... & ... & ... & ... & ...\\ 0 & 0 & ... & q_{i} & l_{i} \end{array}\right]$$

The fundamental matrix is calculated from *M* as $F={\left(I-M\right)}^{-1}$. Entries $F_{\mu \nu }$ represent the expected number of times the system is in state ν, given that the system started at μ. Therefore, $T\left(s_{i}\right)=\sum_{\nu =1}^{C-1}F_{s_{i}\nu }$ throws the expected number of times the system is in a transient state (i.e., its lifespan or mean absorption time). From this quantity, the expected time to collapse for a system with *D* stations starting at state s is obtained as

(18) $$T\left(s\right)=\min\{T\left(s_{i}\right):i=1,\ldots ,D\}.$$

### 5.2. Analytical Limit of the Fundamental Matrix for Random Walks

It is worth exploring the limit of a symmetric probability field: $p_{ij}=1/D^{2}$ involving $p_{i}=q_{i}=\lambda =\left(D-1\right)/D^{2}$. In this case, *F* can be inverted analytically in a simple way (also in the non-random case, but in a much more complicated way [28]).

(19) $$F_{ab}^{r}=\frac{\left(a+b-|b-a|)(2C-a-b-|b-a|\right)}{4C\lambda }.$$

From this expression, the random motion absorption times of station *i* with occupancy $s_{i}$ are given by $T^{r}\left(s_{i}\right)=\sum_{b=1}^{C-1}F_{s_{i}b}^{r}$.

Now, the expected collapse times for random flows given in Equation (18) can be computed as

(20) $$T^{r}\left(s\right)=\frac{1}{2\lambda }\min_{s_{i}}\left\{s_{i}\left(C-s_{i}\right)\right\}.$$

If we define the energy per station as $h\left(s_{i}\right)\equiv {\left(s_{i}-C/2\right)}^{2}$, we can express the expression $T^{r}\left(s\right)$ as

(21) $$T^{r}\left(s\right)=\frac{1}{2\lambda }\left(C^{2}/4-\max\left\{h\left(s_{i}\right)\right\}\right).$$

This way, maximum energies render minimum absorptions, and conversely, for minimum energies, we have maximum absorption times. As expected from our physics analogy, lower energetic states produce larger collapse times.

### 5.3. Imbalance and System’s Performance

Once we have gained some insight into the unbalancing mechanisms, we perform a numerical experiment to estimate the performance of the system in terms of its expected time to collapse. For this, we first prepare a set of non-collapsed states: $\bar{\Omega }\subset \Omega$. Then we apply the procedure described above to estimate the absorption time (i.e., time to collapse) for each state in $\bar{\Omega }$. We repeat the process under different randomization conditions in which the empirical data are shuffled randomly. Notice that for each randomized version of the data, the transition probabilities $p_{ij}$ need to be updated. This way, 0% randomization strength corresponds exactly to the empirical data, while 100% randomization corresponds to a random walk in the simplex with collapse times given by Equation (21). The whole procedure is collected in the algorithms addressed in the Supplementary Material.

**Figure 9:**
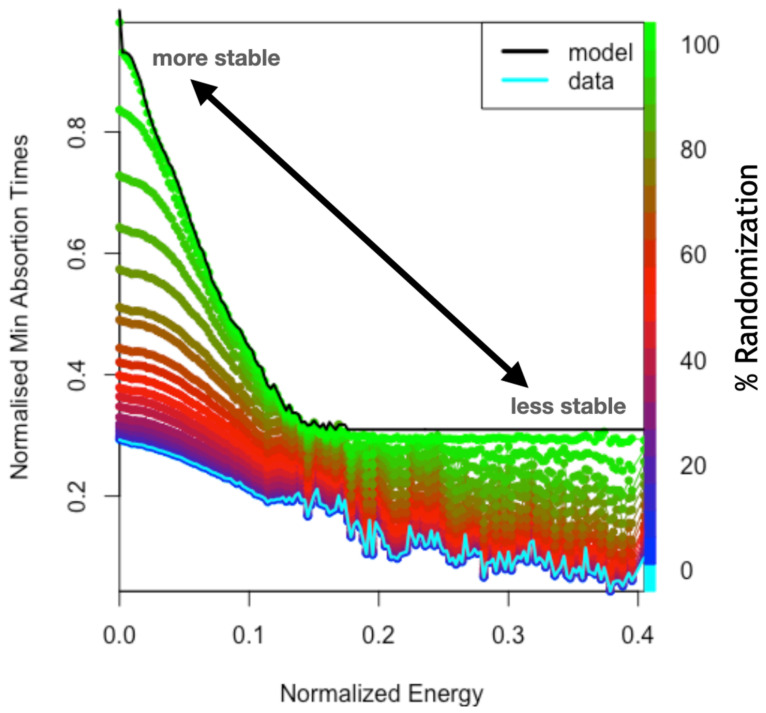
Absorption times: normalised collapse times as a function of normalised energy are shown for different levels of randomisation. Starting from states with a certain energy, their minimum time to collapse is computed. States with higher energy and shorter time to collapse correspond to more unstable situations. The empirical data correspond to 0% random and are shown in cyan. The case of maximum randomisation (light green) corresponds to the model of the collapse times given by Equation (21).

In Figure 9, we show the expected collapse times. States with higher energy and shorter time to collapse correspond to more unstable situations. It should be noted that the performance decreases with increasing imbalance (energy). This decrease is fast up to a certain critical energy (0.15) and then plateaus. Starting from the empirical data (cyan), slightly increasing the random makes the system destabilise faster, decreasing the performance. The observed plateau occurs because of the stiff expression (i.e., the maximum) in Equation (21). As soon as a dimension reaches a certain energy $h^{*}=\max h_{i}$ (that station becomes sufficiently unbalanced), the value of the absorption time will be given by $∼C^{2}/4-h^{*}$. If we keep moving in an unbalance zone $h^{*}$, we will stay at that value by the effect of selecting a maximum among all stations, even if the sum of the total imbalance is larger. Furthermore, the random limit given by Equation (21) reproduced in the figure (black line) is the one that shows a harder transition to the plateau. This is harder because the random limit is an upper bound of the performance.

## 6. Conclusions

The first goal of the paper was to unveil the collapse conditions of a network of shared mobility vehicles. To do so, we built and explored a network of stations with limited capacity through which users commute in shared vehicles. We showed how the dynamics of the system is determined by non-random walks in a certain state space (simplex). In this space, by using statistical physics techniques, it is possible to understand the imbalance of the system in terms of energy, which provides a valuable insight into the diffusion mechanisms. We found that the system tends to collapse naturally over time (which was reported in the highlighted results). In particular, the diffusion is anomalous because the asymmetry of travel trends creates diffusions that are not balanced in different areas of the city. This result explains why an additional external rebalancing mechanism is necessary to keep the system in equilibrium. Furthermore, we gave analytical expressions for the collapse probability and for the super-diffusion exponents.

In light of our model, self-journeys (i.e., loops), although being a small proportion of the total trips, play an important role in the stabilization of the system. The reason is that loops produce no net energy shift in the simplex. Both multiscale entropy and complexity profile techniques have been applied to real data from a bicycle sharing fleet in the city of Salamanca (Spain). The results have shown that the system presents a complexity behaviour that shows self-organisation, which is far from random white noise. This fact is relevant since complex systems are known to have other properties, such as robustness and adaptability. These results can be directly extrapolated to other car-sharing systems.

Furthermore, we believe that our work can be extended in the future to find the seasons and times at which loops have the greatest impact on system stability. These results would help to design mobility policies that promote self-journeys at specific times and in specific areas to maintain system saturation with minimal external intervention. In particular, we characterise the dynamic system and the associated stochastic processes in a way that is independent of the type of vehicles, stations, etc.

It should be noted that our model is limited by the fact that we have neglected many other effects that could lead to system collapse (traffic congestion, loss of vehicles, etc.). However, our analysis, by not taking into account all of these additional factors, provides a conservative limit when investigating a possible real collapse.

To conclude, in future work, we will investigate networks of stations of different capacities as well as the effect of traffic in the system dynamics.

## Data Availability

Not applicable.

## Supplementary Materials

The following are available at https://www.mdpi.com/article/10.3390/e24050606/s1. (A) Derivation of the simplex dimension: we use the methods of Generating Functions to derivate formula in Equation (2), Figure S1. Estimating simplex dimension. An associated counting problem of ways to arrange n balls in k boxes of limited capacity C: (1) a single box with infinite capacity, (2) correction for a finite capacity box, (3) the case of two equal capacity boxes and (4) generalisation to an arbitrary number of boxes. Each panel shows the corresponding sequence and its associated generating function. (B) Pseudo-code for all the algorithms used in our study.
