# Supplementary material for: A Multi-Scale Entropy Approach to Study Collapse and Anomalous Diffusion in Shared Mobility Systems
Source: Entropy (Basel). 2022 Apr 27;24(5):606. doi: 10.3390/e24050606 (PMC9141931; doi:10.3390/e24050606)
Supplement: Supplementary file 1 [file entropy-24-00606-s001.zip › entropy-1680753-supplementary.pdf]

Article

SUPPLEMENTARY MATERIAL for *A Multi-Scale Entropy Approach to Study Collapse and Anomalous Diffusion in Shared Mobility Systems*

Francisco Prieto-Castrillo [1,†,*], Javier Borondo [2,†], Rubén Martín García [3†] and Rosa M. Benito [4,†]

[1] Facultad de Ciencias, Departamento de Matemáticas. Universidad de Oviedo; Calle Federico García Lorca 18, 33007, Oviedo (Asturias), Spain; prietofrancisco@uniovi.es
[2] Facultad de Ciencias Econ. y Empresariales (ICADE), Departamento de Gestión Empresarial. Universidad Pontificia Comillas. Calle Alberto Aguilera 23, 28015 Madrid, Spain; jborondo@comillas.edu
[3] Facultad de Informática. Universidad Pontificia de Salamanca; Calle Compañía, 5, 37002 Salamanca, Spain; rmartinga@upsa.es
[4] Grupo de Sistemas Complejos, E.T.S. de Ingeniería Agron., Alim. y de Biosist., Universidad Politécnica de Madrid, Avda. Puerta de Hierro 2-4, 28040 Madrid, Spain; rosamaria.benito@upm.es
* Correspondence: prietofrancisco@uniovi.es
† These authors contributed equally to this work.

1. Analytical Formula to Estimate the State Space (Simplex) Dimension

Although the derivation of this formula can be done using the methods of Generating Functions (GF) -see e.g. [1]-, we consider that including it in this Supplementary Material may be relevant when classifying points in the simplex.

Say we have k boxes of capacity C in which we want to introduce an arbitrary number of balls (Figure S1). The problem is to compute the number of possible arrangements as a function of these parameters. As stressed, this can be solved with the formalism of the GFs.

Starting with a single box ($k = 1$ in panel 1 of Figure S1) we represent the coefficients of the sequence $(a_n) = (a_0, a_1, \ldots a_n, \ldots)$ as the number of ways to enter a pack of n balls into the box. If such box has unlimited capacity there is only one way to introduce the n-pack: just put all balls inside!

$$(a_n) = (1, 1, 1, \ldots) \sim A(x) = 1 + x + x^2 + x^3 + \ldots = \frac{1}{1-x} \tag{S1}$$

where we have indicated the generating function $A(x) = \sum_{n=0}^{\infty} a_n x^n$ associated to the sequence (a_n). We have also used the geometric series when $|x| < 1$. However, if the box has limited capacity C (panel 2) the possible ways to place the elements are:

$$(\underbrace{1, 1, 1, \ldots, 1}_{C\ times}, 0, 0, \ldots) \tag{S2}$$

where there are C 1's and the remaining quantities are 0. The associated generating function renders: $\tilde{A}(x) = 1 + x + x^2 + \ldots + x^C$.

Now, noting that:

$$\frac{1}{1-x} = \underbrace{1 + x + x^2 + \ldots + x^C}_{\tilde{A}(x)} + x^{C+1}(1 + x + x^2 + \ldots) \tag{S3}$$

we obtain the generating function for a box with capacity C.

$$\tilde{A}(x) = \frac{1 - x^{1+C}}{1-x}. \tag{S4}$$

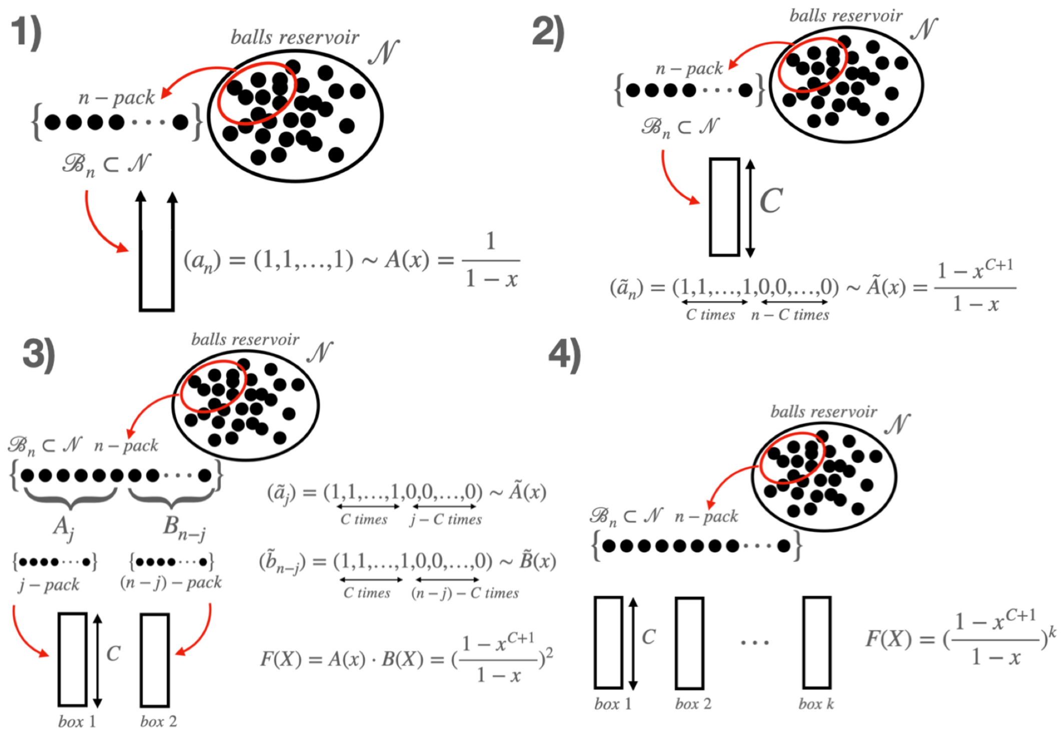

Figure S1. Estimating simplex dimension. An associated counting problem of ways to arrange n balls in k boxes of limited capacity C: 1) a single box with infinite capacity, 2) correction for a finite capacity box, 3) the case of two equal capacity boxes and 4) generalisation to an arbitrary number of boxes. Each panel shows the corresponding sequence and its associated generating function.

If now two C-sized boxes are considered (panel 3), the n-pack can be split into two sub-packs of size j and $n-j$, where $0 \leq j \leq n$. The ways to deploy each pack into each box are simply those previously found for a single C-sized box. However, there are as many as $n+1$ possible partitions of the n-pack now. The compound GF is the product of the corresponding GFs (see [1]). This is nothing else than the convolution rule for generating functions.
Back to the case of having k boxes, we iterate the process just described k times. The resulting GF is:

$$F(x) = [\frac{1-x^{1+C}}{1-x}]^k. \text{ (S5)}$$

The number of possible arrangements formally consists of finding the nth coefficient in the Taylor expansion of $F(x)$ or, in GF notation $[x^n]F(x)$. Using the binomial theorem:

$$F(x) = \sum_{j=0}^{k} \binom{k}{j} (-1)^j x^{j(1+C)} [\frac{1}{1-x}]^k. \tag{S6}$$

Now, using the expansion for $[1/(1-x)]^k$ given by:

$$[\frac{1}{1-x}]^k = \sum_{i=0}^{\infty} \binom{k+i-1}{i} x^i \tag{S7}$$

and substituting above we get

$$F(x) = \sum_{n=0}^{\infty} \sum_{j=0}^{k} (-1)^j \binom{k}{j} \binom{k+n-1}{n} x^{n+j(1+C)} \tag{S8}$$

Doing now the shift $n \to n + j(1+C)$ we obtain:

$$F(x) = \sum_{n=0}^{\infty} x^n [\sum_{j=0}^{k} (-1)^j \binom{k}{j} \binom{k+n-1-j(1+C)}{n-j(1+C)}] \tag{S9}$$

Notice that since n is a non-negative integer $\binom{n}{k} \neq 0 \iff 0 \leq k \leq n$. This way, even if the series coefficients are $\neq 0$ for $n \geq j(1+C)$ one can define the sum starting at $n = 0$ since the binomial coefficients will take care of the cut-off.

2. Algorithms

Algorithm S1 Workflow to compute the Mean Multi-Scale Entropy
INPUT: timestamped Origin-Destination time series $\mathcal{D} = \{(A, B, t)\}$, ns, N,m, r, τ_{max}
OUTPUT: Mean MSE

1: Sort $\mathcal{D}$ by event timestamp t
2: $J \leftarrow$ Buld the trip-trip $(A, B) - (A', B')$ Jaccard distance Matrix using Eq.16 for all possible combinations.
3: $\{\mathbf{u}_k : k = 1, \ldots ns\} \leftarrow$ Generate ns sequential samples of length N with random initial point.
4: **for** $k = 1, \ldots ns$ **do**
5: $MSE(\mathbf{u}_k; m, r, \tau_{max}) \leftarrow$ Compute MSE for $\mathbf{u}_k$ using Alg.2.
6: **end for**
7: **return** $(1/ns) \sum_{k=1}^{ns} MSE(\mathbf{u}_k; m, r, \tau_{max})$

Algorithm S2 Computation of MSE
INPUT: ordered series $\mathbf{u} = (u(1), u(2), \ldots, u(N))$, m, r, τ_{max}
OUTPUT: $MSE(\mathbf{u}; m, r, \tau_{max})$

1: **for** $\tau = 1, \ldots \tau_{max}$ **do**
2: **for** $j = 1, \ldots, \lfloor N/\tau \rfloor$ **do**
3: $\mathbf{y}^{(\tau)} = (y_j^{(\tau)})$ Coarse-grain $\mathbf{u}$ using Eq.15
4: **end for**
5: $SampEn(\mathbf{u}; m, r)_\tau$ Compute $SampEn(\mathbf{y}^{(\tau)}; m, r)$ using Alg.3
6: **end for**
7: **return** $\{SampEn(\mathbf{u}; m, r)_\tau : \tau = 1, \ldots, \tau_{max}\}$

Algorithm S3 Computation of SampEn
INPUT: ordered series $\mathbf{y} = (y_i : i = 1, \dots, L)$ $,m, r$
OUTPUT: $SampEn(\mathbf{y}; m, r)$

for $j = 1 \dots L - m + 1$ **do**
  $v(j) \quad [y_{j+k} : 0 \leq k \leq m - 1]$ {m sized templates}
end for
for $j = 1 \dots L - m$ **do**
  $v^*(j) \quad [y_{j+k} : 0 \leq k \leq m]$ {$m + 1$ sized templates}
end for
$\mathcal{B}_k \quad |\{v(j) : d(v(k), v(j)) \leq r\}|$, $(1 \leq k \leq L - m + 1)$ using Eq.16 for $d(v(k), v(j))$
$\mathcal{A}_k \quad |\{v^*(j) : d(v^*(k), v^*(j)) \leq r\}|$, $(1 \leq k \leq L - m)$ using Eq.16 for $d(v^*(k), v^*(j))$
return $-\log \left(\sum_{k=1}^{L-m} \mathcal{A}_k \Big/ \sum_{k=1}^{L-m} \mathcal{B}_k \right)$

Algorithm S4 Expected Absorption Time for a system's state
INPUT: non-collapsed state $\mathbf{s} = (s_1, s_2, \dots, s_{ne})$, p_{ij}
OUTPUT: $T(\mathbf{s})$

for $i = 1, \dots, ne$ **do**
  $M(i) \leftarrow$ Transition matrix from p_{ij} using Eq.17
  $F(i) \quad (I - M(i))^{-1}$ {Fundamental Matrix}
  $T(s_i) \leftarrow \sum_{b=1}^{C-1} F_{s_i b}$ {lifespan for station i with occupancy s_i}
end for
return $T(\mathbf{s}) \leftarrow \min\{T(s_i) : i = 1, \dots, ne\}$

Algorithm S5 Estimating system's collapse
INPUT: Origin-Destination data $\mathcal{D} = \{(A, B, t)\}$, non-collapsed partial simplex $\overline{\Omega}$
OUTPUT: $[H, T(H)]$ (energy, absorption time list)

$p_{ij} \leftarrow$ Compute (i, j) trips empirical prob. from $\mathcal{D}$
for $\mathbf{s} \in \overline{\Omega}$ **do**
  $u(\mathbf{s}) \leftarrow$ State energy
  $T(\mathbf{s}) \leftarrow$ Absorption Time computed with Alg.4 using $\mathbf{s}$ and p_{ij}
end for
$[H, T(H)] \leftarrow$ with $T(H) = \frac{1}{|\{\mathbf{s}: H(\mathbf{s}) = H\}|} \sum_{\mathbf{s}: H(\mathbf{s}) = H} T(\mathbf{s})$ {Aggregate by state-energy}
return $[H, T(H)]$

Algorithm S**6** Computation of a non-collapsed partial simplex
INPUT: Num. units N, Num. stations D, Station's capacity C, $Nsteps$
OUTPUT: non-collapsed partial simplex $\overline{\Omega}$

$\mathbf{s} \leftarrow$ valid sate in $\Omega = \{\mathbf{s} \in \mathbb{N}^D \mid \sum_{i=1}^{D} s_i = N, 0 \leq s_i \leq C\}$
$\overline{\Omega} = \{\mathbf{s}\}, k \leftarrow 0$
while $k \leq Nsteps$ **do**
    $\mathbf{s}' \quad \mathbf{s}$
    choose i, j randomly from $\mathbf{s}$ such that $s_i > 0$ and $s_j < C$,
    $s'_i \quad s'_i - 1$ in $\mathbf{s}'$ {station i decreases by one vehicle}
    $s'_j \quad s'_j + 1$ in $\mathbf{s}'$ {station j increases by one vehicle}
    $\overline{\Omega} = \overline{\Omega} \cup \{\mathbf{s}'\}$
    $\mathbf{s} \quad \mathbf{s}'$
    $k \quad k + 1$
end while
return $\overline{\Omega}$

3. References

1. Lando, S. *Lectures on Generating Functions*; Student mathematical library, American Mathematical Soc.: Washington, DC, USA, 1955.
